# Risk Perceptions of Cellphone Use While Driving: Results from a Delphi Survey

**DOI:** 10.3390/ijerph15061074

**Published:** 2018-05-25

**Authors:** Motao Zhu, Toni M. Rudisill, Kimberly J. Rauscher, Danielle M. Davidov, Jing Feng

**Affiliations:** 1 The Center for Injury Research and Policy, The Research Institute at Nationwide Children’s Hospital, Columbus, OH 43205, USA; 2 Department of Pediatrics, College of Medicine, Division of Epidemiology, College of Public Health, Ohio State University, Columbus, OH 43210, USA; 3 Department of Epidemiology, School of Public Health, West Virginia University, Morgantown, WV 26506, USA; trudisill@hsc.wvu.edu; 4 Department of Occupational and Environmental Health Sciences, School of Public Health, West Virginia University, Morgantown, WV 26506, USA; krauscher@hsc.wvu.edu; 5 Departments of Emergency Medicine and Social and Behavioral Sciences, West Virginia University, Morgantown, WV 26506, USA; ddavidov@hsc.wvu.edu; 6 Department of Psychology, North Carolina State University, Raleigh, NC 27695, USA; jing_feng@ncsu.edu

**Keywords:** adolescents, young adults, distracted driving, young driver, surveillance

## Abstract

Cellphone use while driving has been recognized as a growing and important public health issue by the World Health Organization and U.S. Center for Disease Control and Prevention. Surveys typically collect data on overall texting while driving, but do not differentiate between various forms of cellphone use. This study sought to improve the survey indicators when monitoring cellphone use among young drivers. Experts and young drivers were recruited to propose behavioral indicators (cellphone use while driving behaviors) and consequential indicators (safety consequences of cellphone use while driving) in 2016. Subsequently, experts and young drivers selected the top indicators using the Delphi survey method. We enrolled 22 experts with published articles on cellphone use while driving nationally, and seven young drivers who were freshmen at a state university. Sending a text or e-mail on a handheld phone was picked as the top behavioral indicator by both groups. However, young drivers chose playing music on a handheld phone as the second most important behavioral indicator, which was overlooked by experts. Injury/death and collision were the top two consequential indicators. Experts and young drivers identified the important survey indicators to monitor cellphone use while driving.

## 1. Introduction

As a major source of morbidity and mortality worldwide, motor vehicle crashes contribute to approximately 20–50 million injuries and 1.2 million deaths annually [1]. Motor vehicle crashes are the leading cause of death among adolescents and young adults worldwide (including the United States (USA)) [2,3,4,5]. Contributing to these crashes is distracted driving, especially cellphone use, which has been recognized as a growing and important public health issue by the World Health Organization (WHO) and the Centers for Disease Control and Prevention (CDC) [6,7]. Epidemiologic studies have clearly established that cellphone use increases a driver’s crash risk [8,9,10,11,12,13,14]. Visual distraction off the road is critical in explaining the risks associated with texting and any other cellphone activities [10,15,16,17]. Sending or receiving a text takes a driver’s eyes off the road for an average of 4.6 s, during which a car traveling 55 miles per hour can travel the length of a football field [18]. Young drivers have reported the highest level of cellphone-related crashes and near crashes, relative to older groups [19,20].

Monitoring the patterns and trends of cellphone use among young drivers is important to provide necessary data for conducting meaningful evaluations and proposing interventions. Today, texting has become one of the most commonly used measures of cellphone use in relation to distracted driving. Two major U.S. surveys (Youth Risk Behavior Survey and Traffic Safety Culture Index) collect information on “overall texting” while driving [21,22], yet overall texting might not be the best measure of cellphone use, because it does not capture any of the various other common forms of cellphone use. In a recent national survey of young drivers, 43% of respondents reported they sent text messages, 12% e-mailed, 21% used the Internet, 12% took videos, 10% engaged in video chats, and 17% took photos/selfies while driving [23]. In addition, respondents said they frequently used social media such as Facebook and Snapchat as well as apps such as YouTube while driving [23]. These findings demonstrate that texting alone might not be sufficient for measuring cellphone use in a young driver population. Therefore, the objective of this study was to identify the most important, and consequential, measures (i.e., indicators) of cellphone use among young drivers in order to better monitor the danger of all types of cellphone use among this high-risk population.

## 2. Materials and Methods 

**Figure 1:**
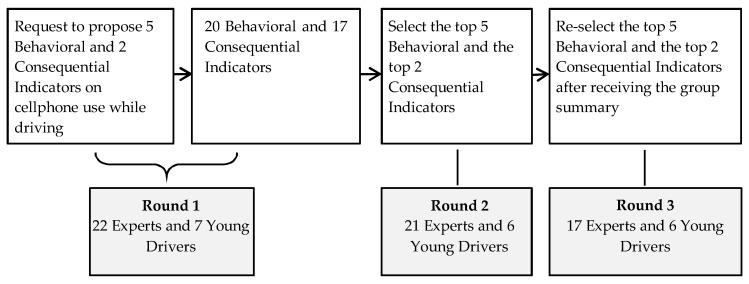
Flow chart: a Delphi Survey on behavioral and consequential indicators of cellphone use while driving, United States, 2016.

We applied the Delphi method, developed by Dalkey and Helmer in the 1950s [24]. The Delphi method is widely used for achieving a convergence of opinions from experts and participants with knowledge and insights on a specific topic [25]. To obtain the views of scientific experts, we recruited the lead or senior authors on articles or reports on cellphone use while driving published between 1 January 2011 and 31 December 2015. To obtain the opinions of young drivers, we recruited freshmen from West Virginia University (18–20 years of age). We sent an e-mail to 42 experts and 30 college freshmen soliciting their participation. Among those invited, 52% of experts (*n* = 22) and 23% of young drivers (*n* = 7) agreed to participate. The Delphi survey was conducted using the web-based survey tool, REDCap [26], and consisted of three rounds (Figure 1).

During the first round, we instructed participants to propose five ways that young people use their cellphones while driving (i.e., behavioral indicators) and two safety consequences of cellphone use while driving (i.e., consequential indicators). Respondents were asked to make the two open-ended lists, irrespective of one another. We summarized the responses from experts and young drivers into 20 unique behavioral and 17 unique consequential indicators. In the second round, we provided all participants with two lists containing the combined responses from experts and young drivers from round 1. We asked them to select from the lists five behavioral indicators and two consequential indicators that they determined to be most important and then provide an explanation for their choices (e.g., dangerousness, commonness, severity). For the third round, we summarized the round 2 responses from experts and young drivers separately, and shared with all participants the frequency of selected indicators and participants’ reasoning for their selection. In this final round, participants were asked once more to select five behavioral and two consequential indicators, and provide a rationale for their selections. 

The study was approved by the West Virginia University Institutional Review Board (1602028919).

## 3. Results

During the first round of the survey, 22 scientific experts and seven young drivers participated. Thirteen (59%) out of 22 scientific experts were female, and most experts were older than 35 years. Nineteen experts were in the United States of America, and the remaining three were in Canada or Australia. There was a good mixture of early-career, middle-career, and senior researchers, and their background was in psychology, public health, medicine, or engineering. Four out of seven young drivers were female, and they were all aged 18–20.

We summarized their responses into 20 unique behavioral indicators and 17 unique consequential indicators of cellphone use while driving (Table 1). Behavioral indicators included calling, texting, navigation, taking photos/videos, gaming, playing music, app use, internet and social media use, checking notifications, looking down while driving, and erratic driving behaviors. Participants also distinguished between handheld vs. hands-free use. Consequential indicators of cellphone use while driving included pedestrian collision, collision avoidance such as sudden braking and swerving, driving errors such as running a red light or stop sign, traffic citations, and collision consequences such as non-fatal injury or death.

**Figure 2:**
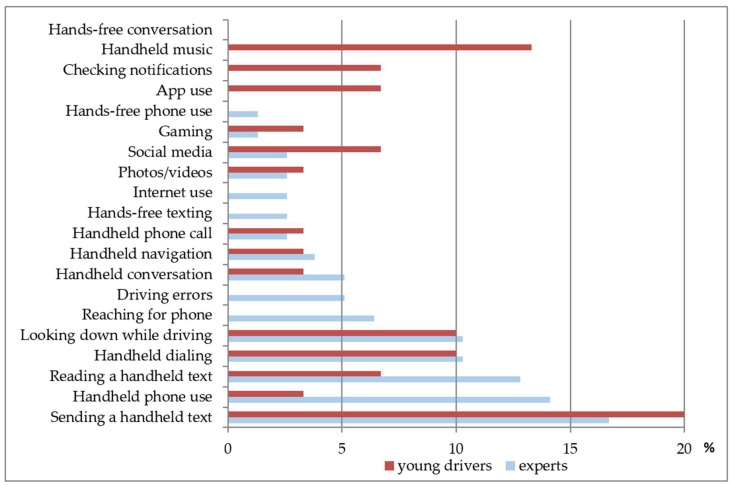
Behavioral indicators of cellphone use while driving presented as a percent of total possible responses from experts and young drivers, United States, 2016.

Figure 2 shows the percentage of round 3 participants (17 experts and six young drivers) who named each behavioral indicator as among the five most important (out of all indicators). Among the experts, the five behavioral indicators selected most often were all related to handheld operations. These included: sending a text or e-mail (17%), handheld phone use (14%), reading a text or e-mail (13%), looking down while driving (10%), and handheld dialing (10%). Among the young drivers, the five behavioral indicators selected most often were sending a handheld text or e-mail (20%), playing music (14%), looking down while driving (10%), handheld dialing (10%), and three measures that tied for 5th place at 7% (checking notifications, social media use, and app use). There were similarities in the indicators selected among both groups; however, playing music accounted for 14% of the young drivers’ selections for the five most important behavioral indicators, while none of the experts had this on their lists. 

Reasons experts proffered for selecting “sending a handheld text” as among the five most important behavioral indicators spanned risk considerations as well as how common they thought this behavior was:

Risk considerations:

- “This requires the driver eyes, hands, and mind to be off the task of driving. Risky, observable and specific.”
- “*Perfect storm: eyes off road, hands off wheel, and mind off road*.”

Commonness:

- “Popular.”

Risk considerations and commonness:

- “*Evidence suggests high level of risk due to complexity of task and need to take eyes off the road repeatedly. Reported prevalence of risk.*”
- “Of the activities involving manipulating a phone, texting also involves considerable visual distraction, cognitive and manual. It is more prevalent than some of the other listed handheld phone uses, and therefore more important.”

Young drivers tendered the following reasons for selecting “sending a handheld text” as among the five most important behavioral indicators: 

Risk considerations:

- “Most dangerous.”
- “It distracts drivers from the road and is the number one reason there are accidents”

Commonness:

- “Because this is the most generic thing that you can do with your phone so naturally it will be the first thing you do in your car while driving.”
- “Texting is a key factor in easy communication nowadays and I think it occurs very often in the car.”

Risk considerations and commonness:

- “A lot of people believe it is still safe, even with all the accidents.”

Although no experts identified playing music on a handheld cellphone as a behavioral indicator, several young drivers selected this as among the five most important indicators for the following reasons:

Commonness:

- “*Most common.”*
- “This is probably the second generic thing you can do in your car because you’re always listening to music, and especially if people are in the car, people will always want you to change it.”
- “Not a lot of people listen to radio anymore.”

Risk considerations and commonness:

- “Aux cords are very popular so a lot of people play their own music off their phones, which can cause them to become distracted while driving.”

**Figure 3:**
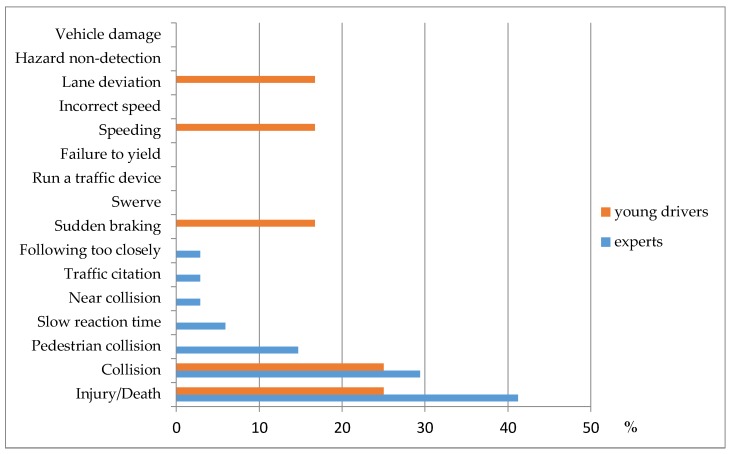
Consequential indicators of cellphone use while driving presented as a percent of total possible responses from experts and young drivers, United States, 2016.

Figure 3 shows the percentage of round 3 participants who named each consequential indicator as among the two most important (out of all indicators). Injury/death and collision were most commonly selected by both experts and young drivers. 

Among both experts and young drivers, the reasons for selecting injury/death focused on severity:

- “*very costly in term of personal pain, suffering, loss, and cost to society*”
- “This is obviously the most serious outcome in the list.”
- “Taking someone’s life or your own, or injuring someone is worst when you were not paying any attention, like while using a cell-phone.”
- *“you cannot reverse a death*.”

The reasoning for selecting collision as among the two most important consequential indicators differed across participant groups. The young drivers cited only issues of severity, while the experts also cited commonness as a consideration. 

Severity:

- “*significant financial, legal and potentially health impacts*”
- “*Collisions are the mechanism for injury and death.*”
- “*pay a lot of money in insurance for causing a collision*”

Commonness:

- “*objective and frequent*”
- “*observable*”

## 4. Discussion

This study sought to identify important behavioral and consequential indicators for assessing cellphone use while driving among youth 18–20 years of age. The purpose was to identify better measures of cellphone use among young drivers that go beyond “texting” in studies of cellphone use while driving. While our results did show that young drivers and scientific experts alike agree that sending a text (or e-mail) on a handheld cellphone is among the five most important behavioral indicators of cellphone use while driving, other cellphone behaviors were also identified as important. These included various handheld cellphone uses such as reading a text (or e-mail), dialing a number, or looking down while driving. 

The identification of texting as an important behavioral indicator in this study is consistent with the literature on the risks of cellphone use while driving. Texting, particularly sending a text or e-mail on a handheld cellphone, is especially dangerous and common among young drivers, according to two national surveys (Traffic Safety Culture Index and Youth Risk Behavior Survey) [22,27]. Relative to reading texts or e-mails while driving, sending texts or e-mails is more dangerous due to the longer time needed for the driver to take their eyes off the road to compose a text message than to glance at an incoming text. Any cellphone activity that requires a driver taking his/her eyes off the road should be avoided [10,15,16,17].

The 2016 Traffic Safety Culture Index, an annual survey of drivers aged 16+ years in the United States, reported that 34% of drivers aged 16–18 years and 59% of drivers aged 19–24 years sent a text or e-mail at least once a month while driving [22]. The 2015 Youth Risk Behavior Survey, a biannual survey among youth in grades 9–12 in public and private schools reported that 42% of high school student drivers sent or read a text message or e-mail at least once a month while driving [27]. Although two national surveys have collected data on texting while driving, these surveys do not include the specific behavioral indicator of sending a text or e-mail on a handheld phone, which the experts and young drivers in our survey deemed as being among the most important behavioral indicators of cellphone use.

In the identification of other indicators besides texting, we found many similarities between experts and young drivers, yet there was one difference worth noting. Young drivers selected playing music as among the most important behavioral indicators of cellphone use while no experts selected this behavior. Young drivers reasoned that playing music was important because it was so common yet this indicator is missing from current national surveys. In the words of one young driver, “*This is probably the second generic thing you can do in your car besides driving because you’re always listening to music, and especially, if people are in the car, people will always want you to change it.*” Young drivers reported using the app Spotify to shuffle through music play lists while driving, or even searching YouTube for a specific song. Another young driver noted, “*Constantly changing music while driving is a huge issue.*” Yet, none of the experts in this study selected playing music on a handheld cellphone as an important behavioral indicator. Generational differences may account for this disconnect. Older experts may be more accustomed to using the radio for their preferred source of music and may not realize that young drivers frequently prefer newer technologies. These technologies allow users to search for favorite songs and compile personalized playlists rather than be limited to trending music on the radio or unwelcome commercial interruptions. Although experts might be comfortable changing music on the radio quickly and without taking their eyes off the road, failing to recognize young drivers’ increasing use of cellphones as a primary source of music overlooks a significant risk indicator in this population. Playing music on a handheld phone involves all three forms of driving distractions: manual distraction to have hand(s) off the wheel, visual distraction when a driver looks down to select songs, and cognitive distraction, particularly if a driver uses YouTube to search for a specific song. Handheld music as a behavioral indicator is also missing from Traffic Safety Culture Index and Youth Risk Behavior Survey surveys. 

Our study also revealed many similarities in the consequential indicators named across participant groups. Both the experts and young drivers selected injury/death and collision as being among the two most important consequential indicators of cellphone use while driving. Experts and young drivers agreed that injury/death can be the most severe consequence of cellphone use while driving. Young drivers selected this item as among the most important because a young driver would not be given a second chance and “cannot reverse a death.” Collisions, which are more common than injury/death, are still a serious consequence. Approximately 6.3 million crashes were reported in the United States in 2015, of which about 32,000 resulted in deaths and 1.7 million resulted in injuries [28], not to mention the costs of vehicle repair and increased insurance premiums for responsible parties in each reported collision. 

### Strengths and Limitations

The main limitation of this study is the limited participation of young drivers. Six young drivers participated in all three rounds of our Delphi survey. While this relatively small number of young drivers may not be representative, their responses generally agreed with those of the 17 scientific experts throughout the USA who also participated in this study, lending them validity. This study is the first to identify behavioral indicators of cellphone use that go beyond texting. 

## 5. Conclusions

Experts and young drivers agree that texting is among the most important indicators of cellphone use, yet they also identified several other behaviors as key indicators. Playing music was chosen as the second most important behavioral indicator by young drivers, but was overlooked by experts. In order to better capture evolving trends of cellphone use while driving and the risk they impose on traffic safety, surveys of young drivers should consider including these additional behavioral indicators. As a result, better data will be available to evaluate the current and develop new policy, education, and other interventions to reduce cellphone use and mitigate the negative consequences caused by cellphone use among young drivers. 

## Figures and Tables

**Table 1:** Behavioral and consequential indicators proposed by experts and young drivers, United States, 2016.

Label	Description
**Behavioral indicators**	
Handheld dialing	A driver holds a cellphone in his/her hand and dials a number
Handheld conversation	A driver holds a cellphone in his/her hand and speaks
Handheld call	A driver answers or makes a call on a handheld cellphone
Reaching for a phone	A driver reaches for a handheld cellphone
Hands-free conversation	A driver talks with hands-free technology
Reading a handheld text	A driver reads a text or e-mail message on a handheld phone
Sending a handheld text	A driver sends a text or e-mail on a handheld phone
Hands-free texting	A driver uses voice to control a phone to send a text or e-mail
Handheld phone use	A drivers uses a handheld phone to call, text, etc.
Hands-free phone use	A driver uses voice to control a phone to call, text, etc.
Handheld navigation	A driver uses a handheld phone to receive navigation instructions
Taking photos/videos	A driver uses a handheld phone to take pictures or videos
Gaming	A driver plays a game with a handheld phone
Handheld music	A driver uses a handheld phone to listen to music
App use	A driver uses apps such as Twitter, Snapchat on a handheld phone
Internet use	A driver searches on the internet with a handheld phone
Social media	A driver checks or updates on social networking sites, e.g., Facebook
Checking notifications	A driver checks for notifications on alerts in general
Looking down while driving	A driver looks down while driving
Erratic driving behaviors	Erratic driving behaviors such as committing lane deviations, driving with variable speed, or reacting slower
**Consequential indicators**	
Near collision	A driver may almost hit another vehicle or object
Collision, non-pedestrian	A driver may collide with another vehicle or object
Pedestrian collision	A driver could hit a pedestrian
Injury/death	A driver could injure or kill another person or him/herself
Sudden braking	A driver may have to suddenly apply the brake to avoid a collision
Swerving	A driver may have to swerve to avoid a collision
Run a red light or stop sign	A driver may run a red light or stop sign
Failure to yield	A driver may proceed without concern for other vehicles or road users
Traffic citation	A driver may receive a traffic citation
Speeding	A driver may not notice a change in speed limit or entrance to school zones
Incorrect speed	A driver may be driving too slowly for road conditions or speed limits
Lane deviation	A driver’s lateral position within the lane may erratically change
Slow reaction time	A driver may respond much slower, e.g., not proceed forward in a timely manner at green lights or stop signs
Hazard non-detection	A driver may not identify critical hazards in the driving environment
Following too closely	A driver may not leave sufficient headway between his/her car and the leading vehicle
Vehicle damage	A driver may damage his/her vehicle or another vehicle
